# Exploring Exercise Interventions for Obstructive Sleep Apnea: A Scoping Review

**DOI:** 10.3390/jfmk10030253

**Published:** 2025-07-02

**Authors:** Irene-Chrysovalanto Themistocleous, Stelios Hadjisavvas, Elena Papamichael, Christina Michailidou, Michalis A. Efstathiou, Manos Stefanakis

**Affiliations:** Department of Health Sciences, University of Nicosia, Nicosia CY-2417, Cyprus; themistocleous.i@unic.ac.cy (I.-C.T.); papamichael.el@unic.ac.cy (E.P.); michailidou.c@unic.ac.cy (C.M.); efstathiou.m@unic.ac.cy (M.A.E.); stefanakis.m@unic.ac.cy (M.S.)

**Keywords:** obstructive sleep apnea, OSA, exercise, exercise training, oropharyngeal exercises, respiratory muscle training

## Abstract

Obstructive sleep apnea (OSA) is the most prevalent sleep disorder caused by breathing difficulties, characterized by repeated episodes of airway blockage while sleeping. Various interventions have been used to improve the symptoms and overall health of individuals with OSA. However, few studies have focused on the impact of exercise on OSA. **Objectives**: The objective of this review was to evaluate the impact of exercise on individuals with OSA, providing an update on the exercise management of OSA. **Methods**: This review examined the current literature, including experimental studies and systematic reviews with meta-analysis, that investigated the impact of exercise (oropharyngeal exercises, respiratory muscle training, and therapeutic exercise training) in OSA patients. Studies were identified by searching databases (PubMed, CHINAL, EBSCO) using the following keywords: obstructive sleep apnea, OSA, exercise, oropharyngeal exercises, respiratory muscle training. Inclusion criteria were based on the PICO framework. **Results**: Forty-three studies were included in this review following the original search, all of which investigated the effects of exercise interventions in OSA. Most of the studies observed various significant health-related improvements following exercise interventions; however, none of them combined or compared all these exercise regimes together. In addition, there is limited information regarding the impact of exercise on sleep architecture. **Conclusions**: Overall, the findings suggest that exercise, regardless of its regime, benefits individuals with OSA.

## 1. Introduction

Obstructive sleep apnea (OSA) is characterized by the repeated collapse of the pharyngeal airway during sleep, resulting in a significant reduction or complete cessation of airflow despite continued breathing attempts [1]. These breathing interruptions lead to imbalances in blood gases, causing hypoxia, hypercapnia, and increased sympathetic activation [1,2]. A common characteristic of OSA is loud snoring, and in many cases, this event is linked to a brief awakening, known as arousal [1]. Another symptom, probably stemming from snoring and arousal, is daytime sleepiness [2]. Furthermore, sleep disorders like OSA can reduce aerobic capacity [3], which restricts activities of daily living [4]. This can be explained by the abnormalities in pulmonary gas exchange during exercise, with increased dead space and systemic vascular dysfunction when resting [5].

These factors may contribute to the greater intensity of dyspnea patients feel during an activity and the limited ability to exercise [5]. Individuals with OSA have a reduction in maximal aerobic capacity as they have demonstrated lower heart rate at peak exercise compared to individuals without OSA, indicating chronotropic incompetence [6]. This chronotropic incompetence has been demonstrated in several studies and it is hypothesized that it may occur due to downregulated beta-adrenergic receptors, which is the result of sympathetic system hyperactivity in individuals with OSA [7,8,9,10,11]. The reduction of maximal aerobic capacity may also be linked to abnormalities in the muscles due to chronic exposure to hypoxia, as individuals with OSA experience bioenergetic changes (upregulated cytochrome oxidase and phosphofructokinase activities), indicating changes in how muscles generate and use energy [12] and structural changes (increased muscle fiber size and protein content), suggesting alterations in muscle physiology [12].

According to international guidelines published in 2017, OSA diagnosis is established via a sleep examination and a polysomnography [13]. These methods define apnea as a 90% reduction in airflow lasting at least 10s and hypoventilation as a decrease in flow of at least 50% coupled with a 3% reduction in oxygen saturation for at least 10s [13]. OSA severity is usually defined by the apnea–hypopnea index (AHI) [13] (Table 1).

The pathophysiological process involved in OSA is complex and includes multiple factors. Additionally, the reasons why OSA can differ significantly among individuals are not well understood [2]. The underling pathophysiological causes of OSA vary among individuals; however, the airway collision involves anatomical and functional factors in the upper respiratory airway, such as the high arch of the palate and hypertrophy of the tonsils, nasal turbinates, and retrognathia, which can lead to airway collapse during sleep [14]. Risk factors for OSA include obesity, age, and gender with additional factors such as family history, ethnicity, and unhealthy lifestyle habits like alcohol consumption and smoking [15,16].

Treating moderate to severe OSA is important because leaving it untreated significantly increases the risk of cardiovascular issues and mortality from all causes [17,18]. On the other hand, treatment for those with mild OSA should be considered if they are symptomatic and experience issues such as insomnia, fatigue during daytime, or sleepiness [19]. The American Academy of Sleep Medicine (AASM) suggests positive airway pressure (PAP) as the preferred treatment for all levels of OSA. Other options apart from continuous positive airway pressure (CPAP) therapy include oral appliances, surgical interventions, and behavioral therapies [19,20]. While CPAP is highly efficient, a significant proportion of individuals with OSA find it difficult to tolerate CPAP, and the acceptance of surgery is low [19,21,22,23]. These findings have prompted the introduction of several new therapies for managing OSA, including exercise as a non-invasive alternative [20,24,25]. Previous studies [26,27,28,29] reported a wide variation of exercise types and programs, as well as multiple combinations of these regimes, making it difficult to assess the impact of exercises in OSA.

The aim of this review is to explore the literature and describe the impact of exercise interventions such as respiratory muscle training (RMT), oropharyngeal exercises (OE), and therapeutic exercise training in individuals with OSA and provide guidance for exercise interventions.

## 2. Materials and Methods

This review included studies on OSA and exercise published in several databases such as PubMed, CHINAL, EBSCO using the following keywords: obstructive sleep apnea, OSA, exercise, oropharyngeal exercises, respiratory muscle training. Studies published in the English language within the last 15 years that met the following Population, Intervention, Comparison, Outcome, Study (PICOS) framework criteria were included in this review: P = adults with OSA; I = physical exercise training, oropharyngeal exercises, or respiratory muscle training; C = any comparison between these interventions, sham or otherwise; O = any OSA health-related parameter; S = clinical trials, pilot studies, case series, systematic reviews, and meta-analyses. Any other type of article, intervention, or different population or age was excluded. Two independent reviewers (I-C.T & S.H) were involved in the screening process of the articles, in which titles and abstracts were screened first, followed by full-text study reports. This scoping review was conducted and reported according to the Preferred Reporting Items for Scoping Reviews (PRISMA-ScR) 2018 statement. The review protocol was prospectively registered on the Open Science Framework (OSF) database with the registration number https://doi.org/10.17605/OSF.IO/C4KJ8 (accessed on 22 May 2025).

## 3. Results

**Figure 1:**
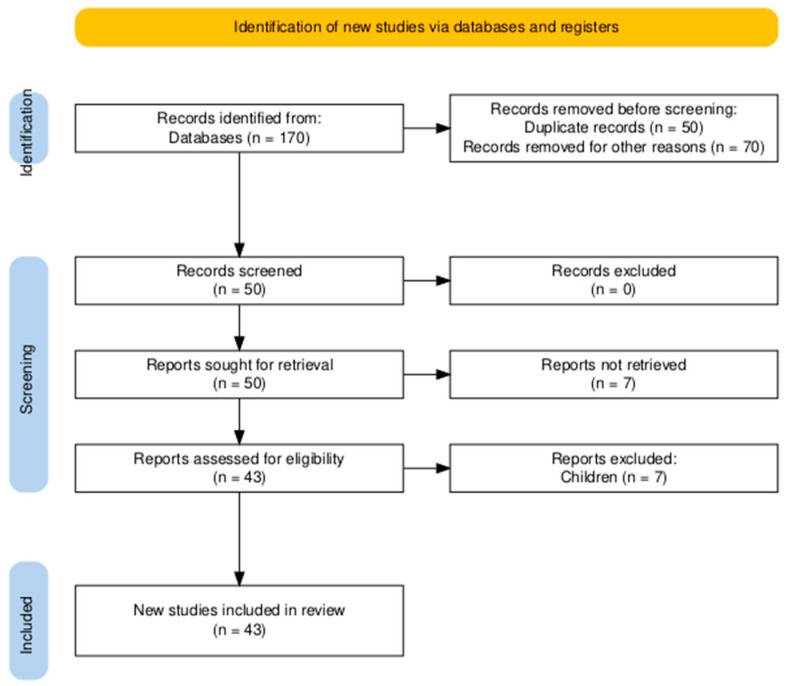
Flowchart diagram.

The initial search identified a total of 170 studies related to OSA and exercise interventions (Figure 1). After screening and following the PICOS framework criteria, 43 studies were included in this review.

These studies were divided into three categories based on the type of exercise studied: RMT, OE, and therapeutic exercise training. The RMT category included 17 studies, the OE category included 12 studies, and the therapeutic exercise category included 13 studies. The study designs varied among these three categories, comprising RCTs, single-arm studies, and systematic reviews with meta-analyses. Table 2 illustrates the number of articles found for each exercise modality as well as a categorization by study type.

### 3.1. Respiratory Muscle Training (RMT)

The upper airway is surrounded by approximately 20 dilator muscles, some of which play a crucial role in stabilizing and dilating the airway during sleep [30]. During wakefulness, the dilator muscles of the upper airway compensate for the anatomical deficits present in patients with OSA. However, during sleep, the activity of the genioglossus (GG) muscle, which is the most well-studied among the dilator muscles, decreases more rapidly in individuals with OSA, leading to upper airway obstruction [31].

There is scientific evidence supporting the application of RMT in individuals with OSA. This training targets the strengthening of muscles involved in breathing, aiming to reduce the collapsibility of the upper airway during sleep [32]. Strengthening of respiratory muscles can be considered a form of pulmonary rehabilitation, as it focuses on enhancing pulmonary function through exercises targeting both the inspiratory and expiratory muscles [20]. Inspiratory muscle training (IMT) involves the use of devices with adjustable resistance and feedback to strengthen the muscles involved in the breathing cycle [33]. IMT involves breathing against resistance several times. To ensure consistency and track repetitions, a percentage of the highest inspiratory pressure of the individual is set by a sensor within the device and a specific number of repetitions are performed with this fixed load [33]. As the muscles become stronger, the resistance level in the device can be increased, allowing for a further improvement in respiratory muscle performance.

The 17 studies on RMT encompass a range of methodological designs, including RCTs [20,34,35,36,37,38,39,40,41,42,43], a single-arm pilot study [44], and systematic reviews [28,29,45,46,47]. While the program of exercise interventions included mostly inspiratory muscle training (IMT), some studies used expiratory muscle training (EMT) [29,40] and others combined various exercise modalities [20,29,42] or non-invasive ventilation (NIV) [29]. The majority of the studies compared IMT to an active control group, given breathing exercises [38], medical treatment [37,41], CPAP [35], IMT without load [36,40], IMT with lower load [41,46,47,48,49], cardiac rehabilitation [29], OE [29], exercise on cycle ergometer [29], EMT [42], and NIV [29]. The remaining studies compared RMT to a non-active control group [20,29,42] or used RMT as a single group [44]. Exercise parameters varied across studies, with frequencies ranging from 2 to 7 days per week and total durations of 2 to 12 weeks. The examined outcome measures also varied among the studies and included the following parameters: AHI; arousal index; mean oxygen saturation (SPO2); oxygen desaturation index (ODI); sleep quality using the Epworth Sleepiness Scale (ESS), the Pittsburgh Sleep Quality Index (PSQI), and the Functional Outcomes of Sleep Questionnaire (FOSQ); maximum inspiratory pressure (MIP); maximum expiratory pressure (MEP); maximal inspiratory mouth pressure (PImax); blood pressure; quality of life (QOL); forced vital capacity (FVC); forced expiratory ventilation in the first second (FEV1); the ratio of the forced expiratory volume in the first second to the forced vital capacity (FEV1/FVC); total lung capacity (TLC); and one repetition maximum (1RM).

The studies in this section present a diverse range of outcomes and highlight the potential benefits of these interventions. RCTs such as those by Arikan et al. (2012) [37] and Andhare et al. (2020) [38] used a threshold device for 7 days a week and 3 days a week compared to medical treatment and breathing exercises, respectively. Both studies reported significant improvements in respiratory muscle strength parameters such as MIP, MEP, 1RM, and sleep quality parameters such as the FOSQ and PSQI post-intervention. It must be noted that while Arikan et al. (2012) [37] reported the exercise parameters and focused on a smaller sample size of 27 participants over 12 weeks without a follow-up, Andhare et al. (2020) [38] had a larger sample size (n = 100) over a shorter 4-week intervention, without clarifying the exercise parameters and without long-term follow-up. These results, due to the small sample size, the lack of long-term follow-up, and the short duration of intervention, limit the generalizability of RMT effects in OSA.

In addition, De Azerado et al. (2022) [35] used a progressive resistive load over a period of 12 weeks and demonstrated significant improvements in MIP, AHI, PSQI, and ESS compared to the active control group (IMT with lower load). It must be considered that while participants (n = 65) were blinded, the intervention was performed once a day without supervision and this potentially affected the adherence to the home-based intervention. Another double-blind study, by Cavalcante-Leao et al. (2024) [42], used IMT and PEP threshold devices for 12 weeks and demonstrated significant improvements in masseter contraction and awakening compared to the control group. While the sample size was extremely small (n = 13) compared to the previous studies and focused on specific physiological measures, which may limit its generalization, it underscores the potential RMT has in respect of other health-related parameters.

Souza et al. (2018) [34] used a 12-week moderate load IMT program on 16 individuals with OSA and reported significant improvements in PSQI but no other significant improvements on functional capacity, lung function, or cardiometabolic parameters. The small sample size, along with the lack of significant changes in the assessed parameters, limits the broader application of this IMT protocol. Ramos-Barrera et al. (2020) [39], on the other hand, highlighted the cardiovascular effects of a high-intensity IMT over 6 weeks, reporting improvements in systolic and diastolic blood pressure, mean arterial pressure, sympathetic nerve activity, and MIP. While these are promising results, suggesting that IMT can be used as a therapeutic approach to manage hypertension in OSA patients, the study’s small sample size (n = 25) and follow-up duration limit its generalizability. In addition, its focus on older, obese individuals in various hypertension categories may not be representative of the entire OSA population.

Another study, by Vranish & Bailey (2016) [43], assessed the effects of IMT (30 breaths each day against a resistance of 75% of PImax) and compared it to a placebo training group (IMT against a resistance of 15% of PImax) for 6 weeks and found reductions in blood pressure, fewer arousals during nighttime, and an improvement in PSQI. However, the results need to be interpreted with caution due to the small sample size (n = 16) and the lack of blood pressure assessment sooner than the end of each week. This neglects the short-term effects and the daily fluctuations in blood pressure. Lin et al. (2020a) [20] assessed a 12-week hospital-based physical therapy program involving two weekly sessions of upper airway muscle strengthening (retropalatal level, retroglossal level, hypopharyngeal level, facial level, and temporomandibular level) for 20 min and RMT for 15 min. Moreover, participants with moderate to severe OSA followed a general endurance training program for 45 min. Participants (n = 15) in the intervention group had a significant improvement in AHI and polysomnography (PSG) outcomes such as arousal index, mean SPO2, and ODI. Despite the improvements, the small sample size and the lack of blinding may lead to bias and limit the generalization of the findings.

Nóbrega-Júnior et al. (2020) [36] found similar benefits with a reduction in AHI, ESS, and PSQI, and improvement in MIP after participants (n = 8) applied IMT twice a day, 7 days per week for 8 weeks in total, without supervision. Similar to previous studies, the small sample size and the lack of supervision during the intervention limit the generalization and the validity of the results. Lin et al. (2020) [41] examined the application of a 12-week IMT program, where one session/week was performed at the hospital and the other four sessions/week were performed at home. Post-intervention participants (n = 16) with moderate to severe OSA demonstrated a significant reduction in AHI and ESS, and improvements in FVC. While this study benefits from its long duration and range of OSA severity categories, it faces some limitations due to the high dropout rate, the small sample size, and the limited supervision during the exercise. A single-arm pilot study by Herkenrath et al. (2018) [44] involved nine males with mild to moderate OSA who underwent a 4-week RMT with a rebreathing bag, 5 days/week. While this protocol demonstrated improvements in SF-36, it failed to show significant improvements in AHI, snoring, or any other PSG parameter, which suggests that this short duration may not be sufficient to improve the symptoms of OSA. The cut-off point seems to be around 4 weeks, as Kuo et al. (2017) [40] used EMT over 5 weeks and reported improvements in AHI, MEP, and PSQI in individuals with moderate OSA. Thus, due to the limited sample size (n = 25) and the lack of improvements in individuals with mild OSA, the application of EMT appears to pose challenges for broader implementation.

Similarly, a review by Torres-Castro et al. (2021) [28] assessed the effects of using physical exercise, OE, and RMT in OSA and revealed significant improvements regarding IMT. Specifically, the three articles that focused on RMT observed improvements in MIP, MEP, and sleep quality, but no improvements in the severity of apnea. It is worth noting that although one of the studies included healthy participants, combining IMT with EMT could provide a more comprehensive intervention and help address the differential effects of these two types of exercise. Dar et al. (2022) [45] verified these findings through a meta-analysis demonstrating significant improvement in PImax, PSQI, ESS, and FEV1 across multiple studies using IMT (at 30–75% of PImax) with a duration of 5–45 min, with a weekly frequency ranging from once a week for 4 weeks and 3 times/week for 12 weeks. Among the seven included studies, only two were scored as having a low risk of bias, as most lacked information regarding their randomization methods. In addition, the studies were heterogeneous as their designs, interventions, and outcomes varied, and their sample sizes were small [45].

Likewise, De Sousa et al. (2024) [29] included 13 studies with significant heterogeneity regarding participants’ characteristics, study design, and exercise intervention. While nine studies compared IMT to a sham and demonstrated improvements in blood pressure parameters and sleepiness, it was reported that IMT was not superior in improving MEP, FVC, FEV1, or AHI. Additionally, this systematic review included some studies comparing IMT with OE, IMT with cardiac rehabilitation, or a combination of IMT with therapeutic exercise training. Nevertheless, they were unable to conduct a subgroup analysis and report more comprehensive results. Cavalcante-Leão et al. (2022) included six studies in their systematic review, where participants followed IMT, EMT, or combined training for 5–16 weeks [47]. The same meta-analysis [47] reported that expiratory exercises demonstrated a decrease in AHI and inspiratory exercises showed a decrease in ESS. In addition, both exercise types demonstrated improvement in the PSQI, although this was not statistically significant in the EMT group. The level of certainty of evidence here [47] was low or very low due to the high heterogeneity between studies. 

A meta-analysis by Chen et al. (2023) [46] evaluated the effects of IMT with high and low intensity and reported significantly lower systolic and diastolic blood pressure following IMT with high intensity compared to the control group. On the other hand, while AHI demonstrated no significant difference between high and low intensity and the control group, PSQI was significantly better in both high and low intensity compared to the control group. In addition, while only low-intensity IMT improved ESS compared to the control group, only high-intensity IMT improved MIP. Finally, neither IMT group affected the ratio of FEV1/FVC% and FVC. In conclusion, the certainty of the evidence in this systematic review was low due to the small sample sizes and concerns regarding the risk of bias.

### 3.2. Oropharyngeal Exercises (OE)

OEs are a non-invasive therapy designed to promote muscle tone in the region of the throat. These exercises target improvements in posture, proprioception, and mobility of the mouth and throat muscles [50]. Specifically, these exercises strengthen the muscles and improve their tone, resulting in the widening of the upper airways during sleep [50]. OE or orofacial myofunctional therapy (OMT) involves isometric and isotonic exercises that target the tongue, the soft palate, and the lateral muscles of the pharyngeal wall [27]. Currently, there are no universally accepted parameters for the exercises or any specific protocol; however, the implementation of OE is entirely dependent on the healthcare professional providing them. Nevertheless, some typical applications focus on tongue elevation and extension in order to encourage a forward resting position of the tongue [51]. In addition, they consist of various exercises targeting the muscles of lips, cheeks, and oropharynx to improve oropharyngeal tone [51]. These applications suggest a frequency of 3-7 days per week, with each session lasting between 15 and 30 min [51]. In this section, 12 studies were included with various methodological designs, including RCTs [52,53,54,55,56], single-arm studies [57,58,59], prospective experimental studies [60], quasi-experimental studies [61], and Cochrane reviews [32]. The comparisons involved OE alone [52,57,58,59,60,61], OE compared with control groups [27,52,53,54,55,56,62], OE compared with other interventions [27,52,62], or OE combined with other interventions [52,62]. The frequency of interventions varied from 2 to 7 times/week and the duration varied from 1 week to 3 months. The examined parameters also varied among the studies, as was the case in the previous section.

An RCT by Ertruk et al. (2020) [27] provides important information regarding the potential use of IMT and OE. Forty-one individuals with OSA were divided into IMT (performed with a threshold loading device 7 days/week), OE (performed exercises 5 days/week), and control groups for 12 weeks in total. Both exercise groups experienced significant reductions in snoring frequency and severity, as well as improvements in fatigue severity and sleep quality. However, no significant changes were found in AHI or sleep quality across the groups. The IMT group demonstrated significant reductions in neck and waist circumference and showed improvements in the strength of respiratory muscles (MIP and MEP) compared to the control-group, while the OE group demonstrated significant increases in the strength of respiratory muscles (MEP) and parameters of sleep (FOSQ), along with a reduction in daytime sleepiness (ESS) compared to the control group. It is essential to note that this study [27] had a high dropout rate and that the exercises were not conducted in a soundproof room, which could impact the outcomes, as external noise may interfere with maintaining the appropriate focus and tone. On the other hand, a prospective experimental cohort study by Verma et al. (2016) [60] reported a significant reduction in neck circumference, daytime sleepiness, apnea, and intensity of snoring following 3 months of OE (5 sets × 10 times of soft palate, tongue, and facial muscles) [60]. Although this study included a small number of participants (n = 20), the exercises were categorized into three phases with graded levels, and the maximum improvement was observed in phase 3 (40–45%). In contrast, while the RCT by Guimarães et al. (2009) [53] reported significant findings in neck circumference, frequency and intensity of snoring, daytime sleepiness, quality of sleep, and AHI post-OE training daily for 3 months, it failed to provide information regarding which set of exercises resulted in maximum benefit for the patients.

Similarly, a quasi-experimental study by Mohamad et al. (2017) [61] found significant improvement in daytime sleepiness, AHI, oxygen desaturation, neck circumference, and snoring index in participants with moderate OSA (n = 15) but not those with severe OSA (n = 15) following OE (tongue, soft palate, facial muscles, and stomatognathic function exercises in 10 min sessions with a frequency of 3–5 times per day) at home. The intervention was performed at home without supervision, and the study lacked information on exercise compliance; however, the results are promising. Another RCT, by Siripajana et al. (2024) [54], used an unsupervised exercise program and reported no significant improvement in the respiratory event index (REI) or the lowest SPO2 within or between the groups (OE vs sham) using the SnoreGym application for a total duration of 10 min. However, the study found changes in lip endurance, anterior tongue strength, and overall endurance following the 2 months of intervention. Although the study was double-blinded, the home sleep tests may not have provided the same level of detail as the lab-based tests used in other studies, which could affect the study’s validity. Moreover, an RCT by Kaur & Mitra (2019) [55] observed significant improvement in neck circumference, snoring frequency, daytime sleepiness, and quality of sleep following 3 months of daily OE (tongue, soft palate, and lateral pharyngeal wall exercises) and a pranayama program lasting for approximately 30 min. The study’s findings are primarily applicable to patients with moderate OSA, while the small sample size necessitates further verification.

Rueda et al. (2020b) [62] in a Cochrane review observed that myofunctional therapy reduced daytime sleepiness compared to sham therapy and may improve the quality of sleep but had variable effects on AHI and snoring compared to CPAP and other interventions. This review included studies with a wide range of populations, such as children, OSA patients, and snorers, which again limits the ability to discern the effects on OSA.

In addition, the certainty of the evidence for all comparisons of this review ranged from very low to moderate. Baz et al. (2012) [57] in a single-arm study found significant improvement in AHI, desaturation parameters including ODI, average duration SaO2 < 90%, % total sleep time, and arousal index following an OMT program (non-articulatory oral myofunctional therapy and articulatory therapy lasting for 10 min 3–5 times per day) twice a week. It is worth noting that the protocol of this study does not provide a detailed description of the exercises and their application, which may have led to variability in execution and affected the reproducibility of consistent results.

An RCT by Diaféria et al. (2017) [52] demonstrated reductions in snoring and sleepiness following a 3-month intervention with OMT (3 daily sessions involving tongue, soft palate, facial muscles, and stomatognathic function exercises lasting for 20 min) alone or in combination with CPAP, along with improvements in tongue and soft palate strength. While the study initially involved 140 participants, only 100 individuals completed the study, indicating a high dropout rate that may have affected the results. In addition, the findings are based on males with OSA, which may not be representative of the entire OSA clinical population. Another RCT by Poncin et al. (2022) [56] reported significant improvements in daytime sleepiness and tongue endurance after a 6-week protocol consisting of tongue elevation exercises (15 min sessions involving three sets of 10 repetitions, while achieving 60% of their maximal elevation force, 4 times per week); however, no significant differences were found in AHI. Nevertheless, the study did not achieve the necessary sample size, which may have affected the statistical power and the applicability of the findings. In addition, previous studies had a protocol that lasted around 3 months, in contrast to this study [56], which had a shorter duration of 6 weeks, and this is probably the reason it failed to demonstrate significant improvement in AHI.

A single-arm study by Younis et al. (2010) [58] found significant improvements in several sleep-related parameters (AHI, arousal index, and the % of total sleep time spent snoring) and desaturation parameters (ODI, average duration of SaO2 <90%, and the % of total sleep time of SaO2 <90%) after a 3-month program consisting of upper airway exercises (tongue and soft palate), applied once a week, with a frequency of 3-5 times per day, for approximately 10 min each time. The small sample size of the study (n = 15), with mild to moderate OSA, limits the extrapolation of the results to the general OSA population. In addition, the protocol was performed without supervision at home and variability in adherence could have affected the outcomes. A pilot single-arm study by Rousseau et al. (2015) [59] found significant improvement in AHI following a 1-week tongue task training regimen, which involved maintaining force within a specific target window for 2 s, followed by a passive recovery period of approximately 8 s, resulting in a task period of 10 s per trial. This cycle was repeated for a total of 360 trials. While the reduction of AHI is promising, the absence of significant improvements in other health-related parameters remains uncertain. Moreover, the small sample size (n = 10) warrants further verification of the results.

### 3.3. Therapeutic Exercise

Exercise and sleep can be beneficial for health and well-being [63]. Individuals with OSA may experience systemic effects that exercise could mitigate, since exercise improves cardiovascular and respiratory function, cognitive and metabolic characteristics, as well as quality of life [64]. Table 3 shows a typical exercise training program that is suggested by Stavrou et al. (2021) [63] as an OSA management strategy.

The therapeutic exercise section included 14 studies, involving RCTs [65,66,67,68,69,70], single-arm studies [71], non-RCT studies [72], and meta-analyses [64,73,74,75,76,77]. Similarly to the previous sections, comparisons involved therapeutic exercise compared with other interventions [64,72,73,75], therapeutic exercise compared with a control group [66,67,68,69,73,74,77], and therapeutic exercise combined with other interventions [65,70,71,76]. The frequency of the programs was 2–3 times per week and they lasted for 4-12 weeks in total. Outcome measure parameters included sleep parameters, quality of life, aerobic capacity, body composition, and respiratory parameters.

Several studies have investigated the effectiveness of exercise on obstructive sleep apnea (OSA), offering valuable insights into potential treatment options. RCTs by Ackel-D’elia et al. (2012) [65] and Sengul et al. (2011) [66] investigated the efficacy of exercise training in OSA, with Ackel-D’elia et al. (2012) showing that CPAP and CPAP combined with exercise (aerobic exercise at 85% of anaerobic threshold (AT) progressing to continuous running above AT for 40 min, supervised 3 times/week) over a 2-month period improved some sleep parameters [65]. The lack of significant differences in most sleep parameters here [65] suggests that the exercise protocol did not directly relate to changes in sleep architecture or respiratory events during sleep. In contrast, the RCT by Sengul et al. (2011) demonstrated significant improvements in AHI and quality of life in the exercise group compared to the control group after a 12-week exercise program involving breathing exercises, warm-up activities, aerobic exercises, and resistance training (low to moderate intensity for the first 2 weeks, progressing to moderate at 60–70% of VO2max) [66]. Nevertheless, the absence of significant differences between the groups may be due to the small sample size (n = 10) in each group, which reduced the statistical power and the ability to detect differences between the two groups. Kline et al. (2011) [67] in an RCT confirmed these results, demonstrating a significant reduction in AHI, along with significant changes in ODI and stage N3 sleep following for 12-week exercise sessions (moderate intensity aerobic exercises at 60% of HRR and resistance training comprising two sets of 10–12 reps for eight different exercises) 4 times/week. Findings based on a single PSG study may not be generalizable to the typical sleep patterns of individuals and can reduce the statistical power to detect true changes resulting from this intervention.

Two RCTs by Karlsen et al. (2017) [69] and Karlsen et al. (2022) [68] reported mixed results regarding the efficacy of high-intensity interval training (HIIT) (4 × 4 min of treadmill running or walking at 90–95% of HRmax 2 times/week) on OSA, with significant improvement in AHI, ESS, and maximal oxygen consumption [VO2peak] in the short term [69], which were not maintained at the longer-term follow-up [68]. Again, there was a single PSG study [69], and the dropout rate in the follow-up study [68] highlights the difficulties of adherence to HIIT protocols, reducing the statistical power of the study and producing difficulties in detecting significant differences between the groups. Neumannova et al. (2018) and Agarwal et al. (2023) [70,72] assessed the combined effects of exercise along with CPAP, with both studies demonstrating significant improvements in various parameters, including neck, waist, and hip circumference, body mass index (BMI), ODI, and pulmonary function. Although the RCT by Neumannova et al. (2018) [70] reported no significant improvements in ODI beyond CPAP alone following pulmonary rehabilitation, it is worth noting that there were significant baseline differences in ODI between the groups, which may have influenced the findings. In the non-RCT by Agarwal et al. (2023) [72], no significant improvements were found in FEV1, similar to previous studies applying IMT [29,46]. Nevertheless, baseline assessment indicated that individuals had normal values.

In a single-arm study by Mittal et al. (2021) [71] improvements in all eight health-related quality of life domains, ESS, BMI, and a 6-minute walk test were found, following a 20-session program of diet and endurance training for upper and lower limbs. The exercise regime included exercises at 50 rpm for 4–6 min, progressing up to 60 min/session with resistance training consisting of 2–4 sets of 10–15 reps using weights [71]. This study did not measure AHI after the end of the intervention; thus, correlating baseline values of AHI with improvements in other parameters only indicates an association, not causation, as other factors may have influenced the outcome.

Meta-analyses by Iftikhar et al. (2014) [64], Peng et al. (2022) [73], Lins-Filho et al. (2019) [74], Olagunju et al. (2022) [75], Bollens & Reychler (2018) [76], and Mendleson et al. (2018b) [77] support the previous findings that exercise interventions, when combined with aerobic and resistance training, can lead to significant improvements in AHI, sleep parameters, VO2peak, and quality of life in individuals with OSA [64,73,74,75,76,77]. These studies reported low to high heterogeneity, which somewhat complicates the interpretation of the results. Finally, a more recent systematic review [46] reported that participating in an exercise program for more than 12 weeks can significantly decrease N2 sleep stage and increase N3.

## 4. Discussion

This review has evaluated the effectiveness of three different exercise modalities in improving outcomes related to obstructive sleep apnea (OSA). When comparing these three exercise types, the results from the reviewed studies are mixed. It seems that RMT, when applied alone, does not lead to improvements in AHI, PSQI, and snoring, but it does significantly affect QOL [44]. In addition, IMT compared with medical treatment [37,41] or a conventional program involving breathing exercises [38] demonstrated improvements in MIP, MEP, FOSQ [37], AHI, ESS, FVC [41], PERF, 1RM, PSQI, and STOP-BANG questionnaire [38]. IMT compared with a non-active control group [20] showed improvements in AHI, SPO2, ODI, and arousal index. The majority of the included articles compared IMT or EMT with a placebo IMT group (sham) and showed improvements in PImax/MIP [35,36], AHI [35,36,40], ESS [35,36,40], PSQI [35,36,40,43], Berlin questionnaire [36], and PEmax [40]. Only one study did not report significant differences between IMT and placebo IMT in lung function parameters and VO2max, but showed significant improvement in PSQI [49]. Additionally, RMT has a significant impact on cardiovascular health. Specifically, IMT compared with placebo IMT (sham) demonstrated improvements in SBP, DBP [39,43], plasma norepinephrine levels [43], and sympathetic nerve activity [39]. Lastly, IMT compared with a non-active control group showed significant improvement in awakening [42] and demonstrated significant difference in masseter contractions between IMT and EMT groups and the non-active control group, in favor of the intervention [42]. These findings from the reviewed studies are consistent with the findings of systematic reviews with meta-analyses [28,29,45,46,47].

Oropharyngeal and tongue exercises demonstrated that OE applied alone improves AHI [59,61], sleep parameters [57,60,61], snoring [58,61], desaturation parameters [57,58,60,61], and arousal index [57,58,60]. In addition, OE compared with a sham group demonstrated improvements in AHI [53], sleep parameters [53,56], snoring [53], neck circumference [53], ESS [54,56], lip endurance [54], tongue strength [54], and endurance [56]. Moreover, when OE was compared with IMT [27] and with a non-active control group [27], both exercise groups demonstrated similar improvements in sleep parameters [27], fatigue [27], and snoring [27]. However, the OE group improved MEP and ESS while the IMT group improved MEP, MIP, and neck circumference [27]. When OMT was compared with sham OMT, CPAP, and CPAP combined with OMT, all groups demonstrated improvements in ESS, AHI, and snoring [54]. However, only OMT and the combined intervention showed improvements in tongue and soft palate muscle strength [52]. Moreover, when OE was combined with pranayama and compared with a sham, it demonstrated improvement in sleep parameters [57], snoring, and neck circumference [55].

Studies comparing exercise interventions with a non-active control group showed improvements in AHI [66,69], ESS [69], aerobic capacity [66,69], QOL, and FSOQ [66]. In contrast, studies on exercise without a comparison group also found improvements in BMI [78], ESS, 6MWT, and HRQOL [78]. In addition, when exercise was compared with medical treatment or with an active control group, it demonstrated improvements in BMI [72], 6MWT [72], ESS [72], SGRQ [72], AHI, ODI, and sleep parameters [67]. Moreover, when exercise was combined with CPAP and compared with a CPAP alone, it demonstrated similar improvements in sleep parameters [65] but showed greater or significant improvements in physical functioning [65]; neck, waist, and hip circumference; BMI; and pulmonary function parameters [70]. Nevertheless, the improvements in the assessed variables were not reported by all studies, probably due to variations in OSA severity; exercise parameters such as frequency, intensity, type of exercise, and duration; sample size; and the lack of power. In addition, some studies lacked supervision during the training, which may have affected the results.

Each one of these exercise interventions targets different mechanisms that could potentially improve the symptoms. While RMT and OE target similar anatomical parts of the body and improve the respiratory muscle strength, they do so in different ways. Specifically, RMT improves the strength of respiratory muscles by breathing against an adjusted resistance, and OE involves exercises for the improvement of tongue position and muscle strengthening of the soft palate and the oropharynx [79]. Nevertheless, while these exercise interventions appear to be more effective in improving several OSA health-related parameters, primarily due to the direct impact on the upper airways, they may be particularly beneficial for OSA individuals with mild to moderate severity, where respiratory mechanics and muscle tone play a significant role. On the other hand, the way in which therapeutic exercise training mitigates the impact of OSA, although not fully understood, is probably not only through weight reduction [80]. Research supports the idea that weight loss can improve AHI over time, but it does not fully explain how therapeutic exercise affects OSA, as body composition parameters do not directly affect AHI [81]. Most likely, therapeutic exercise can improve body composition parameters, leading to the reduction of adipose tissue in the pharyngeal region and upper airway [82]. As mentioned previously, various muscles in the upper airway region play a crucial role in preventing pharyngeal collapse. It is believed that, during exercise, there is an increased activation in respiratory muscle recruitment, which leads to the enhancement of upper airway muscles, thereby increasing the diameter of the airway, reducing its resistance, and preventing pharyngeal collapse during sleep [83,84]. This action improves hypopnea or apnea during sleep. Subsequently, exercise can improve AHI and SaO2 due to improved muscle tone in the upper respiratory tract [73].

In general, it is known that obesity can independently affect the upper airway in various ways, including changes in function and structure, respiratory drive and compensation of load [85]. In addition, obesity related cytokines can affect the neuromuscular regulation of the upper airway [86]. It is important to note that when an individual is exercising regularly, a reduction in AHI might be observed even without substantial weight loss [64,87]. This may be explained by the repeated contraction of the main respiratory muscles during exercise, which leads to an improvement in strength and endurance. These factors may improve the stability of the upper airway and reduce the resistance in the lower airway, leading to fewer events of apnea or hypopnea [83,84]. Another reason for the improvement of AHI with exercise without weight loss is the reduction of inflammatory markers (TNF-α, IL-6) [88]. By reducing these markers, the individual may maintain a more open upper airway and with the production of anti-inflammatory markers there is protection from swelling, allowing the passage of air to remain open for a longer time [89]. All these interrelated factors may explain why exercise, regardless of weight loss, may independently result in AHI improvements.

Moreover, lying down during sleep causes fluid to move and accumulate in the neck, which leads to laryngeal compression that can worsen the severity of OSA [90,91]. Research has found a significant association between decreased lower limb fluid volume and neck circumference. Additionally, a greater movement of fluids from the lower limbs during the night is correlated with a higher AHI, and staying sedentary during the day is associated with an increased movement of fluid in the upper body [92]. Exercise promotes muscle contractions, breathing, and postural changes, which can improve lymphatic function and decrease the accumulation of fluids throughout the body [93,94].

Normal sleep consists of the following stages: (1) rapid eye movement (REM) and (2) non-rapid eye movement (NREM) [84]. NREM is further divided into stages 1, 2, and 3 (slow-wave sleep), which are characterized by deeper sleep, an increased arousal threshold [95], and increased genioglossus activity, which helps to stabilize the airway and prevent it from collapsing [96]. Moreover, increased energy expenditure is shown to be linked to longer periods of N3 sleep [97]. Exercise raises body temperature (due to the metabolic process of muscle contraction) and helps sleep onset by activating mechanisms that diffuse heat and induce sleep [98,99].

Individuals with OSA, due to the lack of oxygen during sleep, may face systemic inflammation, which potentially leads to the progression of inflammatory conditions [100]. OSA can affect the inflammatory process by influencing the release of cytokines from adipose tissue. Elevated levels of inflammatory proteins in individuals with OSA lead to sleepiness, fatigue, and other comorbidities [101]. While exercise is believed to reduce inflammation, the results from these studies suggest that exercise indirectly affects inflammation through its effects on excess fat [89,102]. Nevertheless, there is a limited number of studies examining the effects of different exercise modalities on antioxidant capacity [27].

A study by Fernandes et al. (2022) [103] found that individuals with a mean SPO2 below 95% had higher levels of CRP, leukocytes, and basophils, indicating inflammation. As a conclusion, it was suggested that O_2_ levels are an indication of the level of inflammation. We can hypothesize that in OSA, repeatedly occurring apnea–hypopnea events lead to periods of low O_2_ levels and subsequent reoxygenation, which may induce oxidative stress. Therefore, RMT may improve these periods. The proposed mechanism is that RMT will improve the efficacy of breathing and reduce the periods of apnea–hypopnea due to the reasons stated above, leading to a reduction in low O_2_ and reoxygenation events. These will result in fewer reactive oxygen species (ROS) that lead to the development of biomarkers associated with systemic inflammation [104].

Published studies on various exercise interventions in OSA often focus on AHI, ODI, SPO2, ESS, BMI, neck circumference, and VO2max. To our knowledge, no study to date has assessed the effect of different exercise modalities on parameters such as oxidative stress, disease severity, systemic inflammation, and cardiorespiratory fitness in the OSA population. Nevertheless, a study by Onu et al. (2025) [105] reported that low-intensity exercise (20 < 40% HRR or <3 METs) is beneficial for individuals with chronic conditions, especially those with metabolic syndrome, which often coexists with OSA, due to obesity and insulin resistance [106]. At low intensity, the parasympathetic nervous system is activated, and the hypothalamic–pituitary–adrenal axis (HPA) is reduced, which improves metabolism, boosts antioxidant enzymes, lowers oxidative stress, and enhances total antioxidant status, ultimately improving general cardiovascular health. Due to the physical limitations they face, individuals with OSA may struggle to maintain higher intensities of exercise, but even low-intensity activity like yoga can improve various health care parameters [105,107]. Yoga can help increase adherence to exercise, making it an option for individuals who are unable to exercise intensively due to the severity of their disease. When combined with CPAP, yoga, or tele-yoga can further improve the overall health of the OSA population and improve adherence to CPAP [107].

In addition, moderate-intensity exercise (40 < 60% HRR or 3–6 METs), even without weight loss, can improve lipid profile, VO2max, inflammatory markers, and oxidative stress, as well as body composition parameters [105]. Nevertheless, when this intensity was compared to high intensity, the higher intensity was found superior for antioxidant effects and VO2max [105]. Based on the study by Onu et al. (2025) [105], which included a population that often coexists with OSA, the results of the moderate-intensity exercise are encouraging. A recent meta-analysis found that moderate to high-intensity aerobic exercise is beneficial for individuals with OSA in a dose–response manner [108]. Specifically, performing aerobic training at 50–85% of HRR over 3–5 days per week, appears to offer improvements in AHI, VO2max, and ESS [108]. There are also reports regarding the addition of aerobic exercise to resistance exercise in OSA individuals, suggesting a more beneficial effect [109,110,111] for AHI and VO2max. Resistance exercise has been found to improve fluid accumulation in the lower limbs [112]. Lastly, evidence from a meta-analysis on OSA reported that aerobic exercise, combined exercise, and OE significantly improve several OSA health-related parameters, including AHI [79]. While RMT improved PSQI and ESS, combined exercise was the most effective intervention for lowering AHI [79]. However, to understand how different exercise modalities or intensities affect OSA, further studies targeting this clinical population are necessary.

Regarding the long-term effects of exercise, it was reported that engaging in a consistent exercise training (moderate to high intensity) may improve the activation of upper airway muscles, improve airway diameter, leading to lower airway resistance, reduction of pharynx collapsing [83,84], weight loss [113], and improved ESS [68,114] and ODI [47]. These factors lead to changes that improve apnea–hypopnea periods, suggesting that stronger respiratory muscles enhance AHI.

In order to manage OSA effectively, the exercise intervention must be personalized [48,49]. Considering the existing evidence presented, we suggest the following: Individuals who cannot sustain CPAP and have severe physical limitations may participate in low-intensity aerobic activities. For those who are able to exercise and do not have severe physical limitations, moderate to high intensity aerobic exercise is advised, as it can improve AHI and VO2max even without significant weight loss [108,115]. Considering previous findings, it seems that a longer exercise duration of aerobic training up to 5 days per week over 3 months can help OSA sufferers [108,115]. It is also beneficial to perform resistance training along with aerobic training as it can further benefit individuals with OSA. Additionally, it seems that performing RMT and OE once a week with a frequency of 3–5 times per day, for at least 10 min per session, can improve several health-related parameters. As there is no universally accepted set of exercises and protocols, we suggest up to 5 repetitions per set with 80% to 100% of 1RM to increase muscle strength or 15+ repetitions per set with loads below 60% of 1RM for local muscular endurance [116].

One of the primary limitations of this review is the limited number of studies of each exercise type. In addition, another limitation is that most studies excluded individuals under 18 years old and individuals with co-existing medical diagnoses. Nevertheless, some meta-analyses included in this review involved individuals with comorbid medical conditions, various levels of OSA severity, different age ranges, and of both genders.

## 5. Conclusions

In conclusion, exercise interventions have demonstrated promising effects in individuals with OSA, offering improvements in body composition parameters, OSA health-related parameters, symptoms, and quality of life. While this study was unable to identify one exercise modality that is the most effective, it seems that RMT and OE have the most specific impact, as they can provide better airway stability and, when combined with aerobic and resistance training, can positively affect OSA health-related parameters. However, further research is needed to improve exercise recommendations for the management of OSA and to fully understand the mechanisms by which exercise impacts OSA.

## Figures and Tables

**Table 1:** Apnea–hypopnea index (AHI).

Severity of Apnea	Apnea–Hypopnea Index (AHI)
Normal	<5
Mild	5 ≤ AHI < 15
Moderate	15 ≤ AHI < 30
Severe	≥30

**Table 2:** Articles found for each exercise modality and their categorization by study type.

Exercise Modality	Study Type	Number of Articles
Respiratory muscle training (RMT)	Randomized control trial (RCT)	11
	Systematic review	5
	Single-arm pilot study	1
Orophangeal exercises (OE)	Randomized control trial (RCT)	6
	Quasi-experimental study	1
	Experimental cohort study	1
	Cochrane review	1
	Single-arm study	3
Therapeutic exercise	Randomized control trial (RCT)	6
	Single-arm study	1
	Non-RCT	1
	Systematic review	6

**Table 3:** Aerobic training parameters.

Exercise Parameters (FITT)	Recommendation
Frequency	3–5 days per week
Intensity	**Warm-up/Cool-down:** 50–60% of HRmax **Aerobic program:** Intermittent exercise on 75–85% of HRmax **Strengthening program:** Multi-joint exercise (large muscle groups), 2–8 sets to 6–12 reps on 60–70% of 1RM **Mobility–Flexibility program:** Static or dynamic. Stretch to the point of feeling tightness or slight discomfort, 2–4 sets to 6–12 reps at 10–30 s
Type	Aerobic exercises, strength exercises, mobility-flexibility exercises
Time	45–60 min per session
Duration	3–9 months

**Abbreviations: HRmax:** Heart Rate Maximum; **1RM:** 1 Repetition Maximum.

## Abbreviations

The following abbreviations are used in this manuscript:

OSA	Obstructive Sleep Apnea
AHI	Apnea–Hypopnea Index
PAP	Positive Airway Pressure
CPAP	Continuous Positive Airway Pressure
RMT	Respiratory Muscle Training
OE	Oropharyngeal Exercises
1RM	1 Repetition Maximum
VO2peak	Maximum Aerobic Capacity
ESS	Epworth Sleepiness Scale
ODI	Oxygen Desaturation Index
PSQI	Pittsburgh Sleep Quality Index
BMI	Body Mass Index
METs	Metabolic Equivalent
SPO2	Oxygen Saturation
SGRQ	Saint George Respiratory Questionnaire
FSOQ	Functional Outcome of Sleep Questionnaire
6MWT	6-Minute Walk Test
MIP	Maximum Inspiratory Pressure
MEP	Maximum Expiratory Pressure
PERF	Peak Expiratory Flow Rate
IMT	Inspiratory Muscle Training
EMT	Expiratory Muscle Training
FVC	Forced Vital Capacity
QOL	Quality of Life
HRQOL	Health Related Quality of Life
OMT	Orofacial Myofunctional Therapy
BP	Blood Pressure
SBP	Systolic Blood Pressure
DBP	Diastolic Blood Pressure
FEV1	Forced Expiratory Ventilation in First Second
FEV1/FVC	The ratio of FEV1 in the first second to the FVC
TLC	Total Lung Capacity
NIV	Non-Invasive Ventilation
HRR	Heart Rate Reserve
HRmax	Heart Rate Maximum
